# A Descriptive Study of Spanish and Ecuadorian Commercial Infant Cereals: Are They in Line with Current Recommendations?

**DOI:** 10.3390/nu16131992

**Published:** 2024-06-22

**Authors:** Debby Guevara, Ascensión Marcos, Fátima Isabel Ruiz, Sonia Gómez-Martínez, Susana del Pozo

**Affiliations:** 1 Department of Nutrition and Food Science, Faculty of Pharmacy, Complutense University of Madrid, 28040 Madrid, Spain; debbygue@ucm.es (D.G.); spozocal@ucm.es (S.d.P.); 2 Immunonutrition Research Group, Department of Metabolism and Nutrition, Institute of Food Science and Technology and Nutrition (ICTAN)—CSIC, 28040 Madrid, Spain; amarcos@ictan.csic.es; 3 Retiro Social Services Center, Madrid City Council, 28007 Madrid, Spain; ruizgfa.ext@madrid.es

**Keywords:** Spain, Ecuador, cereals, cookies, infants, toddler, complementary feeding, macronutrients, micronutrients

## Abstract

Cereals are an important source of nutrients, especially used in complementary feeding. The objective of this study is to review the nutritional composition of cereal-based foods for infants from 4 months and toddlers that are offered in Spain and Ecuador, countries selected because of the opportunity to work in them, and due to their socio-economic differences (industrialized and developing countries, respectively). The number of these products was 105 cereals in Spain and 22 in Ecuador. The products were classified as gluten-free cereals, five cereals, eight cereals, multigrain cereals, and cookies. A 25 g serving was used to determine the percentage in which the samples analyzed can cover the Reference Nutrient Intake (RNI) for micronutrients in infants from 7 months and toddlers according to the European Food Safety Authority (EFSA). Nutritional information per 100 g of dry product was collected according to medium, minimum, and maximum units, and nutrient density was calculated. The age range in which these products are recommended is different in both countries. The nutritional composition presents some differences; Spanish cereals show a lower content of sodium, added sugars, hydrolyzed cereals, and maltodextrin than Ecuadorian cereals. Commercialized cereals could contribute to satisfying the nutritional needs of infants and toddlers; however, they can also be a source of non-recommended components.

## 1. Introduction

Diet quality can be influenced by characteristics of the country of residence, such as socio-economic status. Indicators such as Gross Domestic Product (GDP) can give us information about differences between countries. In this sense, Spain and Ecuador, with different GDPs (USD 52.01 thousand per capita and USD 14.48 thousand per capita, respectively), are examples of industrialized and developing countries, respectively [1].

An optimal diet, leading to a nutritional situation that prevents alterations by excess or defect during the first 1000 days of human life (from pregnancy to 2 years of age), is the key to maintaining a good state of health throughout life [2].

Under this premise, the introduction of new foods for “infants”, understood as “children under 12 months of age” [3], is a crucial moment to initiate healthy feeding practices [4]. The introduction of foods other than breastmilk or infant formula as a complement to, rather than a substitute for, breastfeeding is called complementary feeding (CF), or Beikost [5]. According to the current evidence base, the World Health Organisation (WHO) supports the initiation of CF from the sixth month, with particular emphasis on not delaying initiation beyond this time to avoid nutritional deficiencies, especially iron and zinc deficiencies [6]. This initiation of CF at 6 months is also indicated by the Ecuadorian Ministry of Public Health (MSP) [7] and the Spanish Paediatric Association (AEP) [5]. The AEP allows the introduction of foods from 4 months of age in non-breastfed children [5].

The European Society for Pediatric Gastroenterology, Hepatology, and Nutrition (ESPGHAN) also agrees with this recommendation [4]. According to the European Food Safety Authority (EFSA) recommendations, the introduction of foods before 6 months of age is not necessary, except in infants at risk of iron deficiency, when introducing iron-rich foods before 6 months of age could be beneficial [8]. In the LAyDI study, which included 1200 Spanish children born between April 2017 and March 2018, it was observed that the average iron intake was lower than the EFSA recommendations, possibly due to a decrease in the consumption of processed infant foods enriched with iron among children aged 18 to 24 months [9]. Other studies in Spain indicate deficiencies of micronutrients in the diets of more than half of children, particularly in vitamins E and D, calcium, folate, and magnesium [10,11]. Meanwhile, in Ecuador, 9.9% of children under five years of age and 1.8% of school-aged children have iron deficiency [12]. Furthermore, zinc deficiency is present in 27.5% of children under 5 years old and in 28.1% of school-aged children [12].

To initiate this transition between exclusive breastfeeding and Beikost, the latest recommendations of the AEP state that CF can be initiated with any food that does not pose a risk of choking [5], as does the MSP, which suggests starting CF with soft foods [13]. These guidelines in both countries are in line with the infant feeding method called “Baby Led Weaning (BLW)”, which involves the baby leading their own feeding [14], choosing from a variety of soft food offered by the parents/guardians [5,15]. This may lead to better energy self-regulation [16] like the BLISS method, which, in addition to promoting energy self-regulation, aims to prevent iron deficiency by offering foods that meet their nutritional needs [17].

For decades, this initiation of CF has mainly been carried out with the cereal group [18], as it serves as an excellent vehicle for enriching the diet with iron [18], thus covering the needs for this mineral, which are increased in infants from 6 months of age [19]. Cereals are also a good source of phosphorus and potassium, as well as vitamins, including those of the B group, except B_12_ [18,20]. However, it should be remembered that these micronutrients are found in the bran, so their final contribution will depend on the degree of grain processing [18,20]. A frequent practice in the industry is subsequent enrichment [21]. Other reasons for the use of cereals to initiate CF are its mild taste, semi-solid consistency, and texture [22].

All of the above-mentioned benefits led to the popularization of cereal products in infant feeding during the 19th century, resulting in their commercialization [18]. According to the Spanish Agency of Food Safety and Nutrition (AESAN), “Cereal-based foods are those intended to meet the specific needs of infants (children under 12 months of age) and toddlers (children from 1 to 3 years of age) in good health, as a supplement to their diet and/or for their progressive adaptation to the family diet” [3]. The presentation of these products has evolved over time. Initially, infant cereals consisted of a mixture of cereal flour and water. By the mid-19th century, the first infant formulas containing cow’s milk, wheat flour, and malt flour appeared. Subsequently, modified starches became a common component in the preparation of baby foods [18] through the process of hydrolysis, which involves improved starch digestibility and its dispersibility in liquids, resulting in an enhancement of sensory properties, including an increase in sweetness due to the release of sugar [23]. Considering the ESPGHAN recommendations to avoid sugar in CF [4], this practice is not recommended [24]. The European Childhood Obesity Project found that over 95% of Spanish infants aged 9 to 12 months included in its study cohort consumed at least one sugary commercial complementary food [25].

Based on the aforementioned considerations, the aim of this study is to review the nutritional composition of cereal-based foods that are offered to infants from 4 months and toddlers in Spain and Ecuador, countries selected because of the opportunity to work in them and their socio-economic differences (industrialized and developing countries, respectively).

## 2. Materials and Methods

**Figure 1:**
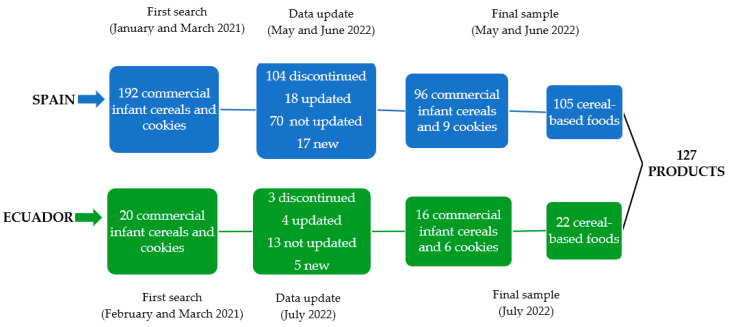
Sample selection.

For the present study, nutritional information and ingredient labeling of 212 processed cereal-based foods were collected in 2021. However, in 2022, the database of this sample was updated to visualize changes in the market of these CF products, incorporating new products and eliminating those that were no longer sold, resulting in a total of 127 processed cereal-based foods. Thus, in Spain, a sample of 192 products was selected between January and March 2021. However, the sample was updated between May and June 2022, resulting in a final sample of 105 products (70 non-updated, 18 updated, and 17 new): 96 commercial infant cereals and 9 cookies (Figure 1). In Ecuador, a sample of 20 products was selected between February and March 2021 and subsequently updated in July 2022, resulting in a sample of 22 products (13 non-updated, 4 updated, and 5 new): 16 commercial infant cereals and 6 cookies (Figure 1). For the selection of these products, researchers included infant cereals for children up to two years of age and infant cookies commercialized in pharmacies, supermarkets, and websites from Spain and Ecuador, excluding cereal-based products for children over two years of age.

The samples were classified using the commercial names of the cereals (gluten-free cereals, 5 cereals, 8 cereals, multigrain cereals, and cookies), as well as the type and number of cereals, mentioning that the “multigrain” category included products that contained more than one cereal, but could not be grouped in either the “5 cereals” or the “8 cereals” categories due to the number of cereals present.

The following data were recorded: the recommended age of consumption by the manufacturer, the types of cereals present in each category, and the number of products with whole grains and added sugars (including glucose, fructose, sucrose, glucose syrup, and those naturally present in honey and fruit juices), using AESAN as a reference [26], and hydrolyzed cereals (when the ingredients indicated hydrolyzed or dextrinized cereal).

The sugar content was examined based on the AESAN criteria, which state that “A food may only be declared as having a low sugar content, as well as any other declaration that may have the same meaning for the consumer, if the product does not contain more than 5 g of sugars per 100 g in the case of solids” [27].

Along these lines, the fiber content indicated on the labeling was also reviewed to determine whether it met the AESAN criteria for declaring a food high in fiber (≥6 g of fiber per 100 g or 3 g of fiber per 100 kcal) [27].

In addition, since the nutritional labeling of the Spanish products only indicated the salt content, the sodium content was calculated by converting salt to sodium (salt in g = sodium in g × 2.5) [28]. However, in Ecuador, this calculation was omitted, as the Ecuadorian products do indicate the sodium content.

Considering the serving size suggested by most manufacturers, a 25 g serving was used to determine the percentage in which the samples analyzed cover the Reference Nutrient Intake (RNI) for vitamins and minerals of infants (7 months to 1 year) and toddlers (1 to 3 years) according to the EFSA [29]. Importantly, the 4- to 6-month-old group was excluded from the analysis due to the absence of commercial cereals targeting children under 6 months in Ecuador, as they are not recommended for this age range [7]. The current study reviewed various nutritional variables, including energy, protein, total fat, saturated fat, carbohydrates, total sugars, and fiber. Additionally, water-soluble vitamins such as vitamin C (ascorbic acid), vitamin B_1_ (thiamin), vitamin B_2_ (riboflavin), vitamin B_3_ (niacin), vitamin B_5_ (pantothenic acid), vitamin B_6_ (pyridoxine), vitamin B_9_ (folic acid), and vitamin B_12_ (cobalamin), as well as fat-soluble vitamins, such as vitamin A (retinol), vitamin D (cholecalciferol), and vitamin E (tocopherol), were reviewed. Minerals such as calcium, iron, zinc, and sodium were also reviewed. For the statistical analysis, nutritional information of these variables was collected per 100 g of dry product according to medium, minimum, and maximum units, and nutrient density (the amount of nutrient/unit of energy, in our case calculated per 1000 kcal) was calculated using Excel 2021.

## 3. Results

### 3.1. Characteristics of the Sample

A total of 127 cereal-based foods were included from Spain (105) and from Ecuador (22). These products were categorized based on their commercial denomination as follows: gluten-free cereals (27), five cereals (13), eight cereals (43), multigrain cereals (29) and cookies (15) (Table 1).

Regarding the recommended age for the consumption of infant cereals, the commercial companies in Ecuador suggest their 22 products from 6 months of age. In contrast, 25 infant cereals from Spain are recommended from 4 months of age, with only one product containing gluten in this group.

### 3.2. Description of Ingredients

The samples were categorized based on the number of cereals they contain, with Spain predominantly featuring products from the “8-cereals” category (n = 39), while Ecuador had products from the “5-cereals” category (n = 6) and “cookies” (n = 6). Additionally, the cereals comprising each category were examined. In both countries, the “gluten-free” category mainly consists of rice and corn. Conversely, the “5-cereals” category is characterized by wheat, barley, oats, and rice, with corn in Ecuador and rye in Spain. The “8-cereals” category typically includes wheat, corn, rice, oats, barley, rye, sorghum, and millet in both countries, also featuring products with triticale and spelt in Spain. In the “multigrain” category, products usually contain wheat, rice, and oats in both countries, while Spain additionally offers products with corn, quinoa, barley, rye, and spelt. Cookies in both countries commonly contain wheat, rice, and oats; meanwhile, often in Spain, corn, barley, and rye are also included.

Table 1 shows the number of products containing gluten, whole grains, added sugars, and hydrolyzed grains. The “5-cereals” category has the highest percentage of products that include whole grains.

It is worth noting that, with the exception of one cookie in Ecuador, all of them mention the presence of added sugars, but none of these products contain hydrolyzed cereals (Table 1).

### 3.3. Energy and Nutrient Content per 100 g of Product

In terms of energy content, children’s cereals offered in Spain (377–438 kcal/100 g) and Ecuador (377–420 kcal/100 g) show similar values. Notably, cookies have the highest caloric content among the categories (Table 2).

Regarding macronutrient content, the samples showed similar amounts of proteins, carbohydrates, and fats. The labeling also indicated the presence of saturated fatty acids, which were found in greater quantities in the cookies compared to the other categories (Table 2).

Following the AESAN criteria [27], 39 products in Spain and 2 products in Ecuador can be classified as “low in sugar” (≤5 g of sugars per 100 g) (Table 2).

The fiber content indicated on the labeling was also examined, revealing that cereals marketed in Spain contain a higher amount (2–8 g/100 g) of fiber compared to those offered in Ecuador (1–4.3 g/100 g), with gluten-free cereals and cookies having the lowest amount of fiber in both countries. Additionally, some products meet the criteria set by AESAN for declaring a food as high in fiber (≥6 g of fiber per 100 g or 3 g of fiber per 100 kcal) [27], despite not explicitly stating it in their commercial name (Table 2).

Regarding the declaration of vitamin content in nutrition labeling, in Ecuador, products in the category of multigrain cereals do not declare the amount of any vitamin and cookies only declare the content of vitamin B_1_. In Spain, on the other hand, multigrain cereals declare the presence of all the vitamins studied and cookies declare vitamins B_1_, B_2_, B_3_, B_5_, B_6_ and C (Table 2).

Among the cereals that report their micronutrient content, Table 2 shows that vitamin C is found in greater quantities in the five-cereal category.

Regarding minerals, the calcium content indicated on the label is higher in the cereals offered in Ecuador. In relation to iron, cereal products and cookies marketed in Ecuador have the highest amount of this mineral. With regard to sodium, cookies are the products with the highest content in both countries (Table 2).

### 3.4. The Nutrient Density of Infant Cereals

To assess the nutritional quality of infant cereals, their nutrient density (amount of nutrients per 1000 kcal) was calculated. Based on this measure, it was observed that the “5-cereals” category exhibits the highest protein density (Table 3).

Regarding fiber density, cereals available in Spain (5–20.7 g/1000 kcal) demonstrate higher levels compared to those in Ecuador (2–11 g/1000 kcal) (Table 3).

From these findings, it is notable that vitamins D and E, in products where they are specified, tend to be higher in cereals from Spain (Table 3).

Based on the minerals most frequently declared in the products reviewed (calcium, iron, zinc, and sodium), we can highlight that, in terms of the nutritional density of iron, the greatest differences between both samples are observed in the “5-cereals” and “cookies” categories, with higher levels in the products commercialized in Ecuador. Additionally, the nutritional density of zinc and sodium was higher in the cereals offered in Ecuador (Table 3).

### 3.5. Results of the Contribution of 25 g of Product to the EFSA Recommended Nutrient Intakes for Infants and Toddlers

Table 4 presents the percentage by which these products can meet the EFSA RNIs for micronutrients of infants (7 months to 1 year) and toddlers (1 to 3 years) in an average serving of 25 g. It is important to note that Table 4 only displays the percentages of RNIs for vitamins and minerals declared in the nutritional labeling. Upon closer examination of the data, it is notable that products in the “5-cereals” category, in a 25 g serving, can meet more than 60% of the RNI for vitamin C. It is worth mentioning that the reviewed sample contributes a higher percentage towards meeting the RNI for vitamin C, while it provides a lower percentage to fulfill the RNI for vitamin B_9_ and sodium.

In terms of the RNI for vitamin A, it can be observed that a 25 g serving of cereals offered in Spain and Ecuador provides more than 39% of the RNI (Table 4).

Considering the percentage contribution of all categories to the RNI for iron, significant differences are noted between the minimum and maximum values in the cereals offered in Ecuador. For instance, products in the “5-cereals” category contribute up to 114% of the recommendations (Table 4).

## 4. Discussion

For the initiation of CF, both countries have developed their own guidelines regarding the age, presentation, and order of introduction of the different food groups [5,7]. These guidelines likely contribute to the notable disparity in the number of cereal-based products identified in Spain (105 products) and in Ecuador (22 products). Moreover, dietary recommendations for children under two years of age in Spain advocate for the consumption of cereals in different formats, including powdered cereals [5], whereas in Ecuador, the emphasis is solely on the consumption of cereals in their natural state [13], possibly influenced by socio-economic and cultural factors. Thus, in another Latin American country similar to Ecuador, such as Chile, a cross-sectional study was developed between August and December 2018 with a sample of 364 mothers of children under 24 months, of whom 11.4% reported having offered cereals as the first food to their infants during the first two years of life [30]. This finding is noteworthy, considering that 12.1% of health professionals in Latin America recommend initiating CF with cereals [31]. In contrast, a Spanish study carried out in 2018, which examined the most commonly used foods among children under two years old, found that 93% of respondents reported using other types of food. This category included cereals such as pasta (62%), bread (53%), semolina (20%), or rice (81%) [32].

According to the results in the current study, in Ecuador, all the cereal products reviewed (22) are recommended by the manufacturer for infants from the age of 6 months, while in Spain, less than a third are indicated for use from 4 months. This finding aligns with the Ecuadorian CF guidelines, which suggests initiating the introduction of foods, including cereals, from 6 months, and the Spanish feeding guide, which, in non-breastfed children, allows the introduction of food from 4 months of age [5,7], similarly to ESPGHAN [4]. The EFSA suggests not introducing food before 6 months unless there is a risk of iron deficiency, recommending in this case the incorporation of iron-rich foods [8]. The ESPGHAN committee notes that around 4 months of age, renal and gastrointestinal functions are mature enough to start CF from week 17 [33]. In contrast, the United States and Mexico recommend introducing cereals from 6 months of age [34,35]. Thus, it only seems to be agreed for the introduction of cereals “not to be ingested before four months of age” [24]. Therefore, further studies are needed to ensure and globally regulate the correct age for introducing cereals into an infant’s diet, considering that the introduction of food requires the development of certain motor skills, physiological processes, and maturity of the various systems in the infant’s body [36].

In relation to the labeling of these products, both countries comply with the mandatory declaration of nutrients and the voluntary declaration of vitamins and minerals. However, in Ecuador, the declaration of sodium is mandatory, while in Spain, the salt content must be declared directly. Below are detailed regulations that must be complied with in each market where infant cereals are commercialized. In Spain, the mandatory nutritional information to be declared in accordance with the provisions of Regulation (EU) No. 1169/2011 of the European Parliament and of the Council of 25 October 2011 includes the following: “The energy value, fats, saturated fats, carbohydrates, sugars, proteins, and salt must be declared “per 100 g or per 100 mL”, which allows comparison between products and, on a voluntary basis, can be declared: monounsaturated and polyunsaturated fatty acids, polyols, starch, dietary fiber, vitamins, or minerals”. This fact explains why cookies in Spain do not include information on all the vitamins and minerals listed in Table 2 and Table 3 [37]. According to the Ecuadorian Technical Standard (NTE) 2618:2013 from the Ecuadorian Institute of Standardization (INEN) for cereal-based foods for infants and young children, the mandatory nutritional information for declaration is as follows: “The energy value, expressed in kilojoules (kJ) or optionally in calories (kcal), and the amount in grams (g) of protein, carbohydrates, and fat per 100 g of food or 100 mL of prepared food and, when appropriate, per serving. As for the declaration of vitamins and minerals, this should be done considering the reference values suggested by the regulations, highlighting that the declaration of these nutrients is not indicated as mandatory” [38]. In addition to the above, the NTE of INEN 1334-2:2011, for the labeling of food products for human consumption, indicates the following: **5.1.2** In addition to the mandatory nutrients, for those products whose total fat content is equal to or greater than 0.5 g per100 g (solids) or 100 mL (liquids), the amounts of saturated fatty acids and trans fatty acids, in grams, shall be declared in addition to the total fat. **5.1.3** The amount of any other nutrient for which a nutrition and health claim is made. **5.1.4** Where a claim is made with respect to the amount or type of carbohydrate, the total amount of sugars should be included, the amounts of starch and/or other carbohydrate constituent(s) may also be indicated. Where a claim is made for dietary fiber content, the amount of dietary fiber should be stated. This regulation also refers to the obligation to declare sodium [39]. All this information implies that the comparison is complicated because it depends on what the manufacturer has included in its label.

Another aspect reviewed in the current study was the type of ingredients, including gluten, which is present in commercial cereals recommended in Spain from 4 months of age and in Ecuador from 6 months of age. In this sense, there is no consensus among all the entities about when to introduce gluten; thus, the MSP of Ecuador recommends including it from 8 months of age [13], while the ESPGHAN suggests avoiding the early (before 4 months) or late (after 7 months) introduction of gluten [40]. On the other hand, the Enquiring About Tolerance (EAT) study found that recruited breastfed infants who consumed enough potentially allergenic foods (including wheat) from 3 months of age had a significant reduction in the prevalence of food allergies [41].

In terms of added sugars, among the samples analyzed, 35 products (33.3%) offered in Spain and 8 products in Ecuador contained added sugars, results similar to those reported in the European Union report based on the Global Novel Products Database, which states that out of 4196 infant foods (including 502 processed cereal-based foods), 1359 products (31.9%) had added or free sugars [42]. Similarly, a study conducted in Africa (Burkina Faso, Cameroon, Ghana, Nigeria, and Senegal) found that 49.4% of commercial baby foods (including cereals) contained added sugars [43]. Considering the request of the ESPGHAN to limit the addition of added sugars to CF products [44], manufacturers should comply with this recommendation and avoid adding any sugars and the hydrolysis process in the manufacture of commercial cereals for infants. This process is unjustified given infants’ ability to digest starch, facilitated by enzymes such as salivary α-amylase and glucoamylase-maltase, which compensate for the deficiency of pancreatic α-amylase typical of their age [45].

In reviewing the number of products containing whole grains, it was established that almost half of the sample in Spain and less than a third of the products in Ecuador contain whole grains. Based on the data presented, we believe that manufacturers should incorporate this type of cereal into their products to contribute to the average fiber requirement, which according to EFSA for children between 1 and 3 years of age is 10 g/day [29]. It is worth emphasizing that in children under one year of age, there are no recommended dietary intakes of fiber, as breast milk (the primary food for this age group) covers their needs [46]. Additionally, studies on dietary habits show the necessity of increasing the consumption of whole-grain cereals in the infant population [47], due to their positive effects on controlling body weight and reducing the risk of diabetes and cerebrovascular diseases [48].

In terms of energy content, among the samples reviewed, “cookies” represent the category that meets the energy content recommended by the Codex Alimentarius, which stipulates that complementary foods consisting of a mixture of cereals should provide no less than 4 kcal/g in dry weight [49]. Moreover, the fat content complies with recommendations, as they contain less than 30% of the total energy from fats and less than 10% from saturated fatty acids, thus aligning with the nutrient profile model of the Pan American Health Organization (PAHO) [50]. However, based on these results, the quantity and quality of energy provided in general may not contribute to weight gain, a significant finding given the high prevalence of childhood obesity in Spain (14.2%) [51] and the double burden of malnutrition in Ecuador, where obesity affects 35.4% of children aged 5–11 [52], while chronic malnutrition affects 20.1% of children under two and 17.5% of children under five [53].

Regarding the presence of carbohydrates, according to AESAN criteria [27], 2 products in the Ecuadorian market and 39 (37%) products in the Spanish market can be classified as “low sugar” infant cereals, a finding similar to another Spanish study conducted in 2020 titled “Current Content of Infant Cereals and Possible Alternatives: Not Everything Counts in Childhood Nutrition”. This study observed a decrease in the sugar content of cereal-based foods, with 18.3% to 30.9% of products with ≤5 g of sugar per 100 g [54]. While these findings indicate a reduction in the sugar content of infant cereals available in Spain, the percentage of products meeting the recommendations remains below 50% in both countries. In our opinion, this result highlights the need to review and modify regulations governing the manufacture and distribution of these foods in order to reduce sugar content.

Taking into account the fact that cereals typically have lower-quality protein compared to animal sources, ESPGHAN suggests that the protein content in cereal porridges should be between 1 and 3 g/100 kcal (excluding those enriched in protein) [55]. The products examined in both countries exhibit an optimal protein content. In terms of vitamin content, this group of products marketed for breastfeeding infants in both countries was found to contribute to meeting the requirements of vitamins such as C, B_6_, and A. In a 25 g serving of product, some categories provide more than 40% of the recommended intakes by EFSA [29], indicating that cereal-based foods in both Spain and Ecuador have great potential to meet the needs of children under 2 years of age. The products examined serve as an important source of vitamin A, covering more than 38% of the needs of infants for this micronutrient, despite not indicating whether they are fortified. According to WHO recommendations, complementary foods can be fortified with micronutrients if necessary [56].

Regarding the recommendations for the intake of micronutrient supplements in the child population, in Spain, specialists often recommend the administration of vitamin D supplements in the form of drops to infants during the first 12 months of life as a preventive measure [57]. In Ecuador, the MSP recommends preventive supplementation with biannual megadoses of vitamin A and providing powdered micronutrients (iron, zinc, vitamin A, folic acid, and vitamin C) to children aged 6 to 24 months as a measure to prevent malnutrition and anemia [58,59]. It is important to highlight that 24.7% of children under five years of age residing in Ecuador receive this micronutrient supplementation to prevent anemia [59]. The OMS also suggests maintaining a varied and fortified diet in the infant population at risk of deficiency to meet vitamin A needs [60]. As reported by a study in Africa (Burkina Faso, Cameroon, Ghana, Nigeria and Senegal), 40.2% of a sample of commercial infant food products (including cereals) were fortified [43], with a higher percentage in Cambodia and Indonesia (72.1% and 65.9% of infant food products were fortified), in contrast to the Philippines, where only 28.4% were fortified with micronutrients [61].

Cereals are also a source of minerals such as zinc, magnesium, iron, and, to a lesser extent, calcium [62]. Regarding sodium content, the reviewed cereal sample meets the Codex Alimentarius specification (recommended sodium values ≤ 100 mg/100 kcal) [63], similar to commercial infant cereals in Germany (27 ± 7.0 mg/100 kcal). However, a Spanish study in 2015 analyzed commercial infant formula and found that they exceeded the maximum allowable level of sodium [64]. We consider that these studies show that the infant feeding industry has sought to adapt its products to the needs of infants, who, due to the immaturity of their kidneys, have a low sodium requirement, making it unnecessary to add salt to complementary feeding [5].

The cereal-based foods reviewed in both countries contribute more than 84% of the iron requirements for the 1–3-year-old age group as indicated by EFSA [29], based on a 100 g serving. In another Latin American country (Honduras), commercial cereals sold in this country were found to have an average content of >4 mg/100 g [65], contributing more than 50% of the EFSA recommendations. Given the importance of this mineral for motor, cognitive, and behavioral development [66], it is essential for cereal-based foods to report their iron content to assess the percentage of the requirement they cover. In Ecuador and Spain, 41% and 62% of products indicate the iron content on the label, whereas in Germany, less than a third of commercial cereals for breastfeeding infants provide this information, highlighting that most of these cereals are fortified [67].

Considering the EFSA zinc requirements [29], in a 25 g serving, the samples from Spain and Ecuador contribute up to 24% and 95% of the requirements for infants between 7 and 12 months. Meanwhile, in countries such as Burkina Faso, Cameroon, Ghana, Nigeria, and Senegal, a medium-sized serving covers between 34% and 58% of the RNI of zinc for infants between 6 and 36 months [43]. It is important to keep in mind that, to meet the needs of infants, fortified foods are an excellent option to avoid diarrhea, poor appetite, growth retardation, and other consequences of zinc deficiency [68].

Limitations of this research include the non-homogeneous sample of products, as the availability of these foods in Ecuador is lower than in Spain. In addition, the nutritional information of infant cereals marketed in Ecuador is not available online, so this study took into account the information provided in stores. Furthermore, due to the dynamic nature of the market for infant products, we had to update the nutritional information of the sample twice, resulting in a decrease in Spain and an increase in Ecuador.

On the other hand, this study is the first to look at the nutritional composition of infant cereals sold in Ecuador, distinguishing it from Spain, where similar analyses have already been carried out.

These studies are highly relevant for evaluating the nutritional quality of complementary foods for breastfeeding infants, given their specific nutritional needs and the potential impact on the health of this age group.

## 5. Conclusions

The recommended age for eating cereal-based foods differs between the two countries. In relation to the description of the nutritional content of cereals marketed in Spain and Ecuador, it shows that this type of product could contribute to meeting the nutritional needs of children under two years of age by being a vehicle for nutrients (carbohydrates, proteins, fats, vitamins, and minerals), mainly fortified products, for the prevention of malnutrition and the development of pathologies related to poor nutrition. In this sense, we consider it necessary to standardize the content of some nutrients such as iron to ensure that needs are met. On the other hand, these products can also be a source of components, such as added sugars, maltodextrin, and/or honey, which are not recommended during the first year of life. It is essential to minimize the content of undesirable components such as those described.

The nutritional composition of the cereals for breastfeeding infants offered in Spain and Ecuador presents some differences; Spanish cereals have a lower content of sodium, added sugars, hydrolyzed cereals, and maltodextrin than Ecuadorian cereals. So far, no studies have been developed in Ecuador to analyze the nutritional quality of infant products. The data obtained in this study may be an adequate starting point to work in this direction, allowing for the development of new research. Meanwhile, in Spain, despite the fact that similar studies have already been conducted, we have been able to update the information and determine the nutritional contribution that the described sample may provide.

## Figures and Tables

**Table 1:** Description of products from Spain and Ecuador.

Commercial Cereal Categories	Country	Total	Gluten	Whole Grains	Added Sugar	Hydrolyzed Cereals
		Number of Products				
**GLUTEN-FREE CEREALS**	**Spain**	**23**	0	6	1	11
	**Ecuador**	**4**	0	0	0	3
**5 CEREALS**	**Spain**	**7**	7	5	0	5
	**Ecuador**	**6**	6	3	1	2
**8 CEREALS**	**Spain**	**39**	39	22	19	21
	**Ecuador**	**4**	4	1	1	3
**MULTIGRAIN CEREALS**	**Spain**	**27**	27	12	6	10
	**Ecuador**	**2**	2	1	1	1
**COOKIES**	**Spain**	**9**	7	1	9	0
	**Ecuador**	**6**	4	1	5	0

**Table 2:** Energy and nutrient content per 100 g of Spanish and Ecuadorian products (median).

	Country	Gluten-Free Cereals	5 Cereals	8 Cereals	Multigrain Cereals	Cookies
**Number of products**	**Spain**	23	7	39	27	9
	**Ecuador**	4	6	4	2	6
		**Median (minimum–maximum)**				
**Energy kcal/100 g**	**Spain**	382 (277–416)	379 (368–388)	377 (366–416)	382 (362–414)	438 (423–466)
	**Ecuador**	380 (380–382)	380 (374–420)	376.5 (370–420)	385 (380–390)	420 (357.1–420)
**Proteins g/100 g**	**Spain**	7.5 (3.9–13.6)	9.6 (5.7–12.4)	9.4 (4.6–14.8)	9.3 (4.6–15.3)	6.8 (1.1–11.2)
	**Ecuador**	7.3 (7–8.5)	10 (7–16)	9.5 (7–15)	8 (6–10)	8 (7.1–10)
* Fats g/100 g	**Spain**	1.5 (0.6–8.1)	1.8 (1.3–4)	2 (0.9–8.6)	2.4 (1.2–8.5)	12.2 (10–14.3)
	**Ecuador**	1.4 (0.5–1.5)	2.5 (1.6–25)	2.2 (1.6–9)	2.8 (1.5–4)	8 (8–10)
** SFA g/100 g	**Spain**	0.2 (0–3)	0.4 (0.2–0.8)	0.4 (0.17–3.1)	0.5 (0.2–3.1)	4.5 (1.2–7.6)
	**Ecuador**	0.3 (0.2–0.5)	0.2 (0–4)	0.3 (0.2–2.9)	1 (1–1)	1 (0.9–1)
**Carbohydrates g/100 g**	**Spain**	83 (72–91)	73.8 (72.8–87.2)	76.5 (68.4–88.5)	76.7 (68–87.1)	75.4 (68.9–81)
	**Ecuador**	84.6 (84–86)	81.5 (68–84)	80.5 (66–84)	83 (82–84)	80 (73–95.2)
* Sugar g/100 g	**Spain**	3.2 (0.3–29)	18 (1.5–28)	20.2 (1–39.1)	7.5 (0.6–35)	24 (12–32.5)
	**Ecuador**	18 (12–28)	22 (1–28)	26.5 (22–37)	23 (22–24)	23 (3–23.8)
**Fiber g/100 g**	**Spain**	2 (0.1–8.5)	8 (1.9–10.5)	6.1 (2.2–11)	6 (1–10.6)	2.3 (0.6–5.3)
	**Ecuador**	1.1 (0.5–5)	4 (2–4)	4.3 (3.3–11)	4.3 (3–5.5)	1 (0.7–4)
		**VITAMINS**				
**Vitamin C mg/100 g**	**Spain**	30 (25–70)	50 (30–71)	30 (30–85)	30 (25–93)	35 (35–35)
	**Ecuador**	42.5 (35–50)	50 (25–60)	35 (25–50)	-	-
**Vitamin B_1_ mg/100 g**	**Spain**	0.5 (0.5–1.6)	0.5 (0.5–0.8)	0.5 (0.5–1.3)	0.8 (0.5–1.8)	0.5 (0.48–1.2)
	**Ecuador**	0.5 (0.4–0.5)	0.5 (0.4–0.5)	0.4 (0.4–0.5)	-	0.8 (0.8–0.8)
**Vitamin B_2_ mg/100 g**	**Spain**	0.6 (0.32–0.6)	0.6 (0.6–0.6)	0.6 (0–0.6)	0.6 (0.32–0.6)	0.6 (0.3–1)
	**Ecuador**	0.6 (0.6–0.6)	0.5 (0.3–0.5)	0.6 (0.3–0,6)	-	-
**Vitamin B_3_ mg/100 g**	**Spain**	6 (3–8.9)	6 (5–6.5)	6 (3–8.5)	7.25 (3–8.9)	5.9 (4.1–10)
	**Ecuador**	5.5 (5–6)	3.6 (3.3–3.6)	5 (3.3–6)	-	-
**Vitamin B_5_ mg/100 g**	**Spain**	2.8 (2–3)	2.8 (2.5–2.8)	2.7 (2–2.8)	2.8 (2–3)	2.4 (1.8–5)
	**Ecuador**	2.9 (2.8–3)	2 (0.2–2)	2.8 (1.5–3)	-	-
**Vitamin B_6_ mg/100 g**	**Spain**	0.4 (0.3–0.8)	0.6 (0.3–0.8)	0.4 (0.25–0.8)	0.4 (0.3–0.8)	0.6 (0.4–1)
	**Ecuador**	0.7 (0.6–0.8)	0.3 (0.3–0.43)	0.6 (0.4–0.8)	-	-
**Vitamin B_9_ µg/100 g**	**Spain**	50 (30–70)	45 (40–56)	50 (30–80)	70 (30–100)	-
	**Ecuador**	52.5 (40–65)	80 (22–80)	40 (22–65)	-	-
**Vitamin B_12_ µg/100 g**	**Spain**	1 (0.5–1.1)	1 (1–1)	1 (1–1.1)	0.5 (0.5–1)	-
	**Ecuador**	1 (0.9–1)	0.7 (0.5–0.7)	0.9 (0.5–1)	-	-
**Vitamin A µg/100 g**	**Spain**	420 (255–450)	435 (300–450)	420 (255–450)	375 (255–450)	-
	**Ecuador**	410 (370–450)	500 (394–500)	394 (370–450)	-	-
**Vitamin D µg/100 g**	**Spain**	7.5 (5–10)	7.5 (7.5–9)	7.5 (5–11.5)	7.5 (5–11)	-
	**Ecuador**	7.3 (7–7.5)	6.6 (6.6–6.6)	7 (6.6–7.5)	-	-
**Vitamin E mg/100 g**	**Spain**	4.4 (2.8–5.4)	4.7 (4.4–5)	4.4 (2.8–6)	4.7 (3–7.3)	-
	**Ecuador**	3.5 (2.5–4.4)	4 (2.4–4)	2.5 (2.4–4.4)	-	-
		**MINERALS**				
**Calcium mg/100 g**	**Spain**	160 (132–678)	256 (145–430)	176.5 (144–669)	179.5 (133–666)	310 (290–329)
	**Ecuador**	324.5 (160–489)	310 (310–435)	420 (160–470)	-	310 (310–310)
**Iron mg/100 g**	**Spain**	7.5 (5.2–8)	7 (5.2–9)	7.5 (2–10.5)	7.5 (5.5–11)	5.9 (5–8.3)
	**Ecuador**	6.5 (6–7)	32 (5.8–31.9)	6 (5.8–7)	-	24 (24–24)
**Zinc mg/100 g**	**Spain**	1.2 (1–4.4)	2.8 (1.1–4.4)	1.2 (1–4.4)	1.3 (1.1–5.3)	-
	**Ecuador**	2.5 (2.5–2.5)	2.8 (2.8–2.8)	2.5 (2.5–2.5)	-	11 (11–11)
* Sodium mg/100 g	**Spain**	16 (8–224)	24 (8–40)	20 (8–240)	16 (8–240)	88 (32–360)
	**Ecuador**	26.5 (12–30)	27.5 (20–150)	32.5 (12–140)	32.5 (30–35)	134.5 (2.5–250)

* Critical nutrients. ** SFA: saturated fatty acid. - No information is provided or the detailed micronutrient composition is not available.

**Table 3:** The nutrient density of the products ***.

		Gluten-Free Cereals	5 Cereals	8 Cereals	Multigrain Cereals	Cookies
**Number of products**	**Spain**	23	7	39	27	9
	**Ecuador**	4	6	4	2	6
		**Median (minimum–maximum)**				
**Proteins g/1000 kcal**	**Spain**	19.8 (9.9–32.7)	25.8 (15–32.5)	25.1 (11.9–35.6)	24.9 (11.9–39.3)	14.6 (2.4–26.3)
	**Ecuador**	19 (18.4–22.4)	25.7 (18.7–38.1)	25.3 (18.9–35.7)	20.9 (15.4–26.3)	20 (19.1–23.8)
**Fiber g/1000 kcal**	**Spain**	5.1 (0.2–22.9)	20.7 (4.9–28.5)	16 (5.5–30.1)	15.7 (2.5–28.8)	5 (1.3–12.1)
	**Ecuador**	2.9 (1.3–13.2)	10.5 (4.8–10.5)	10.7 (8.6–29.7)	11 (7.9–14.1)	2 (2–9.5)
		**VITAMINS**				
**Vitamin C mg/1000 kcal**	**Spain**	79.4 (67–179)	134.6 (77.3–184)	80.4 (72.1–217)	78.1 (65.5–245)	82.2 (82.2–82.2)
	**Ecuador**	112 (91.6–132)	132 (65.8–158)	91.4 (59.5–135)	-	-
**Vitamin B_1_ mg/1000 kcal**	**Spain**	1.5 (1.3–4.2)	1.4 (1.3–2.1)	1.4 (1.3–3.1)	2.1 (1.3–4.9)	1.2 (1.1–2.7)
	**Ecuador**	1.2 (1.1–1.3)	1.2 (1.1–1.3)	1 (1–1.4)	-	2 (1.9–1.9)
**Vitamin B_2_ mg/1000 kcal**	**Spain**	1.6 (0.8–1.6)	1.6 (1.6–1.6)	1.6 (0.8–1.6)	1.6 (0.8–1.7)	1.4 (0.7–2.3)
	**Ecuador**	1.6 (1.6–1.6)	1.3 (0.9–1.3)	1.6 (0.8–1.6)	-	-
**Vitamin B_3_ mg/1000 kcal**	**Spain**	16 (7.2–30.7)	16.2 (12.9–16.8)	16 (7.2–22.7)	19 (7.3–24.6)	13.9 (9.7–23.4)
	**Ecuador**	14.4 (13.1–15.8)	9.5 (8.7–9.5)	13.1 (7.9–16.2)	-	-
**Vitamin B_5_ mg/1000 kcal**	**Spain**	7.2 (4.8–8)	7.5 (6.4–7.6)	7 (4.8–7.7)	7.4 (4.8–8.3)	5.6 (4.1–11.8)
	**Ecuador**	7.6 (7.4–7.9)	5.3 (0.6–5.3)	7.6 (3.6–7.8)	-	-
**Vitamin B_6_ mg/1000 kcal**	**Spain**	0.9 (0.7–2.2)	1.6 (0.8–2.2)	0.9 (0.6–2.2)	1.1 (0.7–2.1)	1.4 (0.9–2.3)
	**Ecuador**	1.8 (1.6–2.1)	0.8 (0.8–1.1)	1.6 (1–2.2)	-	-
**Vitamin B_9_ µg/1000 kcal**	**Spain**	129 (72.1–253)	119 (108–145)	130 (72.1–204)	185 (72.5–264)	-
	**Ecuador**	138 (105–170)	211 (58–211)	108 (52.4–170)	-	-
**Vitamin B_12_ µg/1000 kcal**	**Spain**	2.7 (1.3–2.7)	2.7 (2.7–2.7)	2.7 (2.6–2.7)	1.3 (1.2–2.6)	-
	**Ecuador**	2.5 (2.4–2.6)	1.8 (1.3–1.8)	2.4 (1.2–2.7)	-	-
**Vitamin A µg/1000 kcal**	**Spain**	1084 (613–1354)	1159 (775–1223)	1097 (613–1230)	993 (616–1187)	-
	**Ecuador**	1076 (969–1184)	1316 (1037–1316)	966 (938–1216)	-	-
**Vitamin D µg/1000 kcal**	**Spain**	20 (12–36.1)	20.2 (19.3–23.3)	20.1 (12–29.3)	19.8 (0–29)	-
	**Ecuador**	19 (18.3–19.7)	17.4 (17.4–17.4)	18.3 (15.7–20.3)	-	-
**Vitamin E mg/1000 kcal**	**Spain**	11.7 (7.2–13.8)	12.4 (11.8–13.2)	11.9 (7.2–15.1)	12.2 (7.3–19.1)	-
	**Ecuador**	9 (6.5–11.6)	10.5 (6.3–10.5)	6.5 (5.7–11.9)	-	-
		**MINERALS**				
**Calcium mg/1000 kcal**	**Spain**	425 (336–1630)	679 (374–1156)	455 (363–1617)	477 (345–1609)	708 (678–778)
	**Ecuador**	853 (419–1287)	816 (816–1145)	768 (0–1135)	-	738 (738–738)
**Iron mg/1000 kcal**	**Spain**	19.5 (12.5–27.1)	19 (13.7–23.3)	19.8 (5.3–26.8)	20 (13.3–28.8)	13.9 (11.4–19.6)
	**Ecuador**	17.1 (15.7–18.4)	84 (15.3–84)	14.7 (0–18.9)	-	57 (57.1–57.1)
**Zinc mg/1000 kcal**	**Spain**	3.1 (2.7–11.5)	7.2 (2.8–11.6)	3.1 (2.5–11.7)	3.3 (2.8–13.7)	-
	**Ecuador**	6.5 (6.5–6.5)	7.4 (7.4–7.4)	6.5 (6.5–6.5)	-	26 (26.2–26.2)
**Sodium mg/1000 kcal**	**Spain**	42.2 (21.1–539)	64.5 (21.1–109)	51.7 (20.8–577)	43.2 (20.1–580)	208 (69.7–845)
	**Ecuador**	69.7 (31.4–78.9)	73 (52.6–357)	87.8 (67.6–67.6)	84.4 (79–89.7)	345 (6–595)

*** Nutrient density/1000 kcal. - No information is provided or the detailed micronutrient composition is not available.

**Table 4:** Results of the contribution of 25 g of product to the EFSA Recommended Nutrient Intakes for infants and toddlers.

	Age	Country	Gluten-Free Cereals	5 Cereals	8 Cereals	Multigrain Cereals	Cookies
			VITAMINS				
**Vitamin C (%)**	**7–12 month**	**Spain**	38	63	38	38	44
		**Ecuador**	53	63	44	-	-
	**1–3 years**	**Spain**	38	63	38	38	44
		**Ecuador**	53	63	44	-	-
**Vitamin B_1_ (%)**	**7–12 months**	**Spain**	42	42	42	67	42
		**Ecuador**	42	42	33	-	67
	**1–3 years**	**Spain**	25	25	25	40	25
		**Ecuador**	25	25	20	-	40
**Vitamin B_2_ (%)**	**7–12 months**	**Spain**	38	38	38	38	38
		**Ecuador**	38	31	38	-	-
	**1–3 years**	**Spain**	25	25	25	25	25
		**Ecuador**	25	21	25	-	-
**Vitamin B_3_ (%)**	**7–12 months**	**Spain**	34	34	34	41	34
		**Ecuador**	31	20	28	-	-
	**1–3 years**	**Spain**	20	20	20	24	20
		**Ecuador**	19	12	17	-	-
**Vitamin B_5_ (%)**	**7–12 months**	**Spain**	23	23	23	23	20
		**Ecuador**	24	17	23	-	-
	**1–3 years**	**Spain**	18	18	17	18	15
		**Ecuador**	18	13	18	-	-
**Vitamin B_6_ (%)**	**7–12 months**	**Spain**	33	50	33	33	50
		**Ecuador**	58	25	50	-	-
	**1–3 years**	**Spain**	17	25	17	17	25
		**Ecuador**	29	13	25	-	-
**Vitamin B_9_ (%)**	**7–12 months**	**Spain**	0.02	0.02	0.02	0.02	-
		**Ecuador**	0.02	0.03	0.01	-	-
	**1–3 years**	**Spain**	0.01	0.01	0.01	0.01	-
		**Ecuador**	0.01	0.02	0.01	-	-
**Vitamin B_12_ (%)**	**7–12 months**	**Spain**	17	17	17	8	-
		**Ecuador**	17	12	15	-	-
	**1–3 years**	**Spain**	17	17	17	8	-
		**Ecuador**	17	12	15	-	-
**Vitamin A (%)**	**7–12 months**	**Spain**	42	44	42	38	-
		**Ecuador**	41	50	39	-	-
	**1–3 years**	**Spain**	42	44	42	38	-
		**Ecuador**	41	50	39	-	-
**Vitamin D (%)**	**7–12 months**	**Spain**	19	19	19	19	-
		**Ecuador**	18	17	18	-	-
	**1–3 years**	**Spain**	13	13	13	13	-
		**Ecuador**	12	11	12	-	-
**Vitamin E (%)**	**7–12 months**	**Spain**	22	24	22	24	-
		**Ecuador**	18	20	13	-	-
	**1–3 years**	**Spain**	12	13	12	13	-
		**Ecuador**	10	11	7	-	-
			**MINERALS**				
**Calcium (%)**	**7–12 months**	**Spain**	14	23	16	16	28
		**Ecuador**	29	28	38	-	28
	**1–3 years**	**Spain**	9	14	10	10	17
		**Ecuador**	18	17	23	-	17
**Iron (%)**	**7–12 months**	**Spain**	17	16	17	17	13
		**Ecuador**	15	73	14	-	55
	**1–3 years**	**Spain**	27	25	27	27	21
		**Ecuador**	23	114	22	-	86
**Zinc (%)**	**7–12 months**	**Spain**	10	24	10	11	-
		**Ecuador**	22	24	22	-	95
	**1–3 years**	**Spain**	7	16	7	8	-
		**Ecuador**	15	16	15	-	64
**Sodium (%)**	**7–12 months**	**Spain**	2	3	2.5	2	11
		**Ecuador**	3	3.4	4	4	17
	**1–3 years**	**Spain**	0.4	0.5	0.5	0.4	2
		**Ecuador**	0.6	0.6	0.7	0.7	3

- No information is provided or the detailed micronutrient composition is not available.

## Data Availability

The raw data used in this study are available from the corresponding author upon request due to Privacy.

## References

[1] International Monetary Fund GDP per Capita, Current Prices. https://www.imf.org/external/datamapper/PPPPC@WEO/OEMDC/ADVEC/WEOWORLD.

[2] Moreno Villares J.M., Collado M.C., Larqué E., Leis Trabazo M.R., Sáenz de Pipaon M., Moreno Aznar L.A. (2019). The First 1000 Days: An Opportunity to Reduce the Burden of Noncommunicable Diseases. Nutr. Hosp..

[3] Agencia Española de Seguridad Alimentaria y Nutrición (AESAN) Alimentos Para Grupos Específicos de Población. https://www.aesan.gob.es/AECOSAN/web/para_el_consumidor/ampliacion/productos_dieteticos.htm.

[4] Fewtrell M., Bronsky J., Campoy C., Domellöf M., Embleton N., Mis N.F., Hojsak I., Hulst J.M., Indrio F., Lapillonne A. (2017). Complementary Feeding: A Position Paper by the European Society for Paediatric Gastroenterology, Hepatology, and Nutrition (ESPGHAN) Committee on Nutrition. J. Pediatr. Gastroenterol. Nutr..

[5] Gómez M. (2018). Recomendaciones de La Asociación Española de Pediatría Sobre Alimentación Complementaria. https://www.aeped.es/sites/default/files/documentos/recomendaciones_aep_sobre_alimentacio_n_complementaria_nov2018_v3_final.pdf.

[6] Organización Mundial de la Salud (OMS) (2010). La Alimentación del Lactante y del Niño Pequeño.

[7] Tello B., Gutiérrez P., Caicedo R., Mena A., Cuadrado F., Fernández F., Jarrín E., Coronel E., Vinueza K., Aguirre A., Ministerio de Salud Pública (2015). Paso a Paso por una Infancia Plena.

[8] European Food Safety Authority (EFSA) (2019). Age to Start Complementary Feeding of Infants.

[9] Gómez-Martín M., Domínguez B., Gueimonde M., González S. (2021). Identification of Nutritional Targets in Spanish Children Belonging to the LAyDI Cohort for the Development of Health Promotion Strategies in the First Two Years of Life. Int. J. Environ. Res. Public Health.

[10] Cuadrado-Soto E., López-Sobaler A.M., Jiménez-Ortega A.I., Aparicio A., Bermejo L.M., Hernández-Ruiz Á., Villoslada F.L., Leis R., de Victoria E.M., Moreno J.M. (2020). Usual Dietary Intake, Nutritional Adequacy and Food Sources of Calcium, Phosphorus, Magnesium and Vitamin D of Spanish Children Aged One to <10 Years. Findings from the EsNuPi Study. Nutrients.

[11] López-Sobaler A.M., Aparicio A., González-Rodríguez L.G., Cuadrado-Soto E., Rubio J., Marcos V., Sanchidrián R., Santos S., Pérez-Farinós N., Dal Re M.Á. (2017). Adequacy of Usual Vitamin and Mineral Intake in Spanish Children and Adolescents: ENALIA Study. Nutrients.

[12] Freir W.B., Ramírez-Luzuriaga M.J., Belmont P., Mendieta M.J., Silva-Jaramillo K., Romero N., Sáenz K., Piñeiros P., Gómez L.F., Monge R. Encuesta Nacional de Salud y Nutrición ENSANUT-ECU 2012. https://www.ecuadorencifras.gob.ec/documentos/web-inec/Estadisticas_Sociales/ENSANUT/MSP_ENSANUT-ECU_06-10-2014.pdf.

[13] Ministerio de Salud Pública (MSP) Normas y Protocolos de Alimentación Para Niños y Niñas Menores de 2 Años. https://www.salud.gob.ec/wp-content/uploads/2019/07/4_alimentacion_ni%C3%B1o_menor_2a%C3%B1os.pdf.

[14] D’Auria E., Bergamini M., Staiano A., Banderali G., Pendezza E., Penagini F., Zuccotti G.V., Peroni D.G. (2018). Baby-Led Weaning: What a Systematic Review of the Literature Adds On. Ital. J. Pediatr..

[15] Rapley G. (2011). Baby-Led Weaning: Transitioning to Solid Foods at the Baby’s Own Pace. Community Pract..

[16] Ministry of Health (MOH) Baby-Led Weaning—Te Whatu Ora Position Statement. https://www.tewhatuora.govt.nz/health-services-and-programmes/nutrition/baby-led-weaning-te-whatu-ora-position-statement.

[17] Daniels L., Heath A.-L.M., Williams S.M., Cameron S.L., Fleming E.A., Barry J.T., Wheeler B.J., Gibson R.S., Taylor R.W. (2015). Baby-Led Introduction to SolidS (BLISS) Study: A Randomised Controlled Trial of a Baby-Led Approach to Complementary Feeding. BMC Pediatr..

[18] Núñez-Ramos R., Moreno-Villares J.M. (2019). Los Cereales en la Alimentación del Lactante y el Niño Pequeño. Acta Pediatr. Esp..

[19] Fernández-Palacios L., Ros G., Frontela C. (2015). Nutrientes Clave en la Alimentación Complementaria: El Hierro en Fórmulas y Cereales. Acta Pediatr. Esp..

[20] Gil Hernández Á., Martín-Lagos R.A., Ruiz López M.D., Editorial Médica Panamericana (2017). Tratado de Nutrición Composición y Calidad Nutritiva de los Alimentos.

[21] Latham M.C., FAO (2002). Nutrición Humana en el Mundo en Desarrollo.

[22] Sakashita R., Inoue N., Tatsuki T. (2003). Selection of Reference Foods for a Scale of Standards for Use in Assessing the Transitional Process from Milk to Solid Food in Infants and Pre-School Children. Eur. J. Clin. Nutr..

[23] Fernández-Artigas P., Guerra Hernández E., García-Villanova B. (2001). Changes in Sugar Profile during Infant Cereal Manufacture. Food Chem..

[24] Klerks M., Bernal M.J., Roman S., Bodenstab S., Gil A., Sanchez-Siles L.M. (2019). Infant Cereals: Current Status, Challenges, and Future Opportunities for Whole Grains. Nutrients.

[25] Theurich M.A., Zaragoza-Jordana M., Luque V., Gruszfeld D., Gradowska K., Xhonneux A., Riva E., Verduci E., Poncelet P., Damianidi L. (2020). Commercial Complementary Food Use amongst European Infants and Children: Results from the EU Childhood Obesity Project. Eur. J. Nutr..

[26] Agencia Española de Consumo, Seguridad Alimentaria y Nutrición (AECOSAN) Plan de Colaboración la Mejora de la composición de los Alimentos y Bebidas Otras Medidas. https://www.aesan.gob.es/AECOSAN/docs/documentos/nutricion/PLAN_COLABORACION_2020.pdf.

[27] Agencia Española de Seguridad Alimentaria y Nutrición (AESAN) Tabla Declaraciones Nutricionales Autorizadas 2019. https://www.aesan.gob.es/AECOSAN/docs/documentos/seguridad_alimentaria/gestion_ries-gos/Tabla_declaraciones_NUTRICIONALES_autorizadas.pdf.

[28] Diario Oficial de la Unión Europea Comunicación de La Comisión. Sobre Preguntas y Respuestas Relativas a la Aplicación del Reglamento (UE) n.o 1169/2011 del Parlamento Europeo y del Consejo Sobre la Información Alimentaria Facilitada al Consumidor. https://eur-lex.europa.eu/legal-content/ES/TXT/PDF/?uri=CELEX:52018XC0608(01).

[29] Calleja C.A., Hurtado C., Daschner Á., Fernández Escámez P., Manuel C., Abuín F.-C., María R., Pons G., Fandos E.G., José González Muñoz M. Informe del Comité Científico de la Agencia Española de Seguridad Alimentaria y Nutrición (AESAN) Sobre Ingestas Nutricionales de Referencia para la Población Española. https://www.aesan.gob.es/AECOSAN/docs/documentos/seguridad_alimentaria/evaluacion_riesgos/informes_comite/INFORME_RECOMENDACIONES_DIETETICAS.pdf.

[30] Quintiliano-Scarpelli D., Lehmann N., Castillo B., Blanco E. (2021). Infant Feeding and Information Sources in Chilean Families Who Reported Baby-Led Weaning as a Complementary Feeding Method. Nutrients.

[31] Ladino L., Vázquez-Frias R., Montealegre L., Bagés-Mesa M.C., Ochoa-Ortiz E., Medina-Bravo P.G. (2022). E-1500: Survey on Feeding Practices in the First 1,500 Days of Life, Recommended by Healthcare Professionals in Latin America. Rev. Gastroenterol. Mex..

[32] Zamanillo-Campos R., Rovira-Boixadera L., Rendo-Urteaga T. (2021). Common Practices and Beliefs in the Preparation of Complementary Infant Feeding in a Spanish Sample: A Cross-Sectional Study. Nutr. Hosp..

[33] Grupo de Gastroenterología Pediátrica Zona sur Oeste de Madrid Alimentación del Lactante y del Niño de Corta Edad. https://www.ampap.es/wp-content/uploads/2019/04/Alimentacion-del-lactante-y-del-ni%C3%B1o-de-corta-edad.pdf.

[34] Jana L.A., Shu J. Cereal in a Bottle: Solid Food Shortcuts to Avoid. https://www.healthychildren.org/English/ages-stages/baby/feeding-nutrition/Pages/Cereal-in-a-Bottle-Solid-Food-Shortcuts-to-Avoid.aspx.

[35] Secretaria de Gobernación NORMA Oficial Mexicana NOM-043-SSA2-2012, Servicios Básicos de Salud. Promoción y Educación para la Salud en Materia Alimentaria. Criterios para Brindar Orientación. https://www.dof.gob.mx/nota_detalle.php?codigo=5285372&fecha=22/01/2013#gsc.tab=0.

[36] Jiménez Ortega A.I., Martínez García R.M., Velasco Rodríguez-Belvis M., Ruiz Herrero J. (2017). From Infant to Child. Nutr. Hosp..

[37] Parlamento Europeo y del Consejo Reglamento (UE) No 1169/2011 del Parlamento Europeo y Del Consejo. https://www.boe.es/doue/2011/304/L00018-00063.pdf.

[38] (2013). Elaborados a Base de Cereales para Lactantes y Niños Pequeños. Requisitos.

[39] (2011). Rotulado de Productos Alimenticios para Consumo Humano. Parte 2. Rotulado Nutricional. Requisitos.

[40] Ribes-Koninckx C., Dalmau-Serra J., Moreno Villares J.M., Diaz Martín J.J., Castillejo de Villasante G., Polanco Allue I. (2015). La Introducción Del Gluten En La Dieta Del Lactante. Recomendaciones de Un Grupo de Expertos. An. Pediatría.

[41] Perkin M.R., Logan K., Bahnson H.T., Marrs T., Radulovic S., Craven J., Flohr C., Mills E.N., Versteeg S.A., van Ree R. (2019). Efficacy of the Enquiring About Tolerance (EAT) study among infants at high risk of developing food allergy. J. Allergy Clin. Immunol..

[42] European Commission (2019). Feeding Infants and Young Children.

[43] Khosravi A., Bassetti E., Yuen-Esco K., Sy N.Y., Kane R., Sweet L., Zehner E., Pries A.M. (2023). Nutrient Profiles of Commercially Produced Complementary Foods Available in Burkina Faso, Cameroon, Ghana, Nigeria and Senegal. Nutrients.

[44] Fidler Mis N., Braegger C., Bronsky J., Campoy C., Domellöf M., Embleton N.D., Hojsak I., Hulst J., Indrio F., Lapillonne A. (2017). Sugar in Infants, Children and Adolescents: A Position Paper of the European Society for Paediatric Gastroenterology, Hepatology and Nutrition Committee on Nutrition. J. Pediatr. Gastroenterol. Nutr..

[45] Lin A.H.M., Nichols B.L. (2017). The Digestion of Complementary Feeding Starches in the Young Child. Starch-Stärke.

[46] Edson Bustos A., Alexis Medina P. (2020). Recommendations and Effects of Dietary Fiber for Children. Rev. Chil. Nutr..

[47] Fundación Iberoamericána de Nutrición (2020). International Life Sciences Institute Papel de los Cereales de Grano Entero en la Salud.

[48] Aparicio A., Salas-González M.D., Lorenzo-Mora A.M., Bermejo L.M. (2022). Nutritional and Health Benefits of Whole Grains Cereals. Nutr. Hosp..

[49] Organización de las Naciones Unidas para la Alimentación y la Agricultura (FAO), Organización Mundial de la Salud (OMS) Directrices sobre Preparados Alimenticios Complementarios para Lactantes de Más Edad y Niños Pequeños. https://www.fao.org/fao-who-codexalimentarius/sh-proxy/pt/?lnk=1&url=https%253A%252F%252Fworkspace.fao.org%252Fsites%252Fcodex%252FStandards%252FCXG%2B8-1991%252FCXG_008s.pdf.

[50] Organización Panamericana de la Salud (OPS), Organización Mundial de la Salud (OMS) Modelo de Perfil de Nutrientes de la Organización Panamericana de la Salud. www.paho.org/permissions.

[51] Gómez S., Lorenzo L., Ribes C., Homs C., Gasol Foundation (2019). Resultados principales del estudio PASOS 2019 sobre la actividad física, los estilos de vida y la obesidad de la población española de 8 a 16 años. Estudio PASOS.

[52] Instituto Nacional de Estadística y Censos Encuesta ENSANUT. https://www.ecuadorencifras.gob.ec/documentos/web-inec/Estadisticas_Sociales/ENSANUT/ENSANUT_2018/Principales%20resultados%20ENSANUT_2018.pdf.

[53] Instituto Nacional de Estadística y Censos (INEC) Principales Resultados Nacional sobre Desnutrición Infantil—ENDI. https://www.ecuadorencifras.gob.ec/documentos/web-inec/ENDI/Presentacion_de_Resultados_ENDI_R1.pdf.

[54] Vitoria Miñana I. (2021). Current Content of Infant Cereals and Possible Alternatives: Not Everything Counts in Childhood Nutrition. Anales de Pediatria.

[55] Gómez-Álvarez Salinas P. (2002). Papillas de Cereales. Recomendaciones.

[56] Organización Mundial de la Salud (OMS) Alimentación Del Lactante y Del Niño Pequeño. https://www.who.int/es/news-room/fact-sheets/detail/infant-and-young-child-feeding.

[57] Asociación Española de Pediatría Vitaminas. https://enfamilia.aeped.es/vida-sana/vitaminas.

[58] Ministerio de Salud Publica (MSP) (2011). Normas, Protocolos y Consejería para la Suplementación con Micronutrientes.

[59] Tello B., Rivadeneira M.F., Rubio-Codina M., Parra J., Medina D. Reportes de la ENSANUT 2018. https://www.ecuadorencifras.gob.ec/documentos/web-inec/Bibliotecas/Libros/Reportes/Reportes_ENSANUT_Vol1_Salud_de_la_Ninez.pdf.

[60] World Health Organization (WHO) (2011). Guideline: Vitamin A Supplementation in Infants and Children 6–59 Months of Age.

[61] Bassetti E., Zehner E., Mayhew S.H., Nasser N., Mulder A., Badham J., Sweet L., Crossley R., Pries A.M. (2022). Nutrient Profiles of Commercially Produced Complementary Foods Available in Cambodia, Indonesia and the Philippines. Public Health Nutr..

[62] Carbajal Azcona Á. Manual de Nutrición y Dietética. https://www.ucm.es/data/cont/docs/458-2018-01-10-cap-14-alimentos-2018.pdf.

[63] Organización Mundial de la Salud (OMS), Organización de las Naciones Unidas para la Alimentación y la Agricultura (FAO) Norma para Alimentos Elaborados a Base de Cereales para Lactantes y Niños Pequeños. https://www.fao.org/fao-who-codexalimentarius/sh-proxy/en/?lnk=1&url=https%253A%252F%252Fworkspace.fao.org%252Fsites%252Fcodex%252FStandards%252FCXS%2B74-1981%252FCXS_074s.pdf.

[64] Mir-Marqués A., González-Masó A., Cervera M.L., De La Guardia M. (2015). Mineral Profile of Spanish Commercial Baby Food. Food Chem..

[65] Fernández-Palacios L., Ros-Berruezo G., Barrientos-Augustinus E., Jirón de Caballero E., Frontela-Saseta C. (2017). Aporte de Hierro y Zinc Bioaccesible a la Dieta de Niños Hondureños Menores de 24 Meses. Nutr. Hosp..

[66] Organización Panamericana de la Salud (OPS), Organización Mundial de la Salud (OMS) La Importancia del Hierro un Desarrollo Saludable. https://www3.paho.org/hq/dmdocuments/2011/Iron_Nutrition%20SPA.pdf.

[67] Theurich M.A., Koletzko B., Grote V. (2020). Nutritional Adequacy of Commercial Complementary Cereals in Germany. Nutrients.

[68] National Institutes of Health Datos sobre El Zinc. https://ods.od.nih.gov/pdf/factsheets/Zinc-DatosEnEspanol.pdf.

